# Heterologous production of the insecticidal pea seed albumin PA1 protein by *Pichia pastoris* and protein engineering to potentiate aphicidal activity via fusion to snowdrop lectin *Galanthus nivalis* agglutinin; GNA)

**DOI:** 10.1186/s12934-023-02176-1

**Published:** 2023-08-17

**Authors:** Jake S. De-Thier, Prashant Pyati, Jack Bell, Jennifer J. Readshaw, Adrian P. Brown, Elaine C. Fitches

**Affiliations:** 1 School of Biosciences, University of Durham, Durham, DH1 3LE UK; 2 Plant Biotechnology Research Centre, Ajeet Seeds Pvt. Ltd, Aurangabad, 431133 India; 3 FUJIFILM Diosynth Biotechnologies Billingham, Billingham, TS23 1LH UK

**Keywords:** Pea seed albumin, Pa1b, Snowdrop lectin, Fusion protein, *Pichia pastoris*, V-ATPases aphid, Bumble bee

## Abstract

**Background:**

New bioinsecticides with novel modes of action are urgently needed to minimise the environmental and safety hazards associated with the use of synthetic chemical pesticides and to combat growing levels of pesticide resistance. The pea seed albumin PA1b knottin peptide is the only known proteinaceous inhibitor of insect vacuolar adenosine triphosphatase (V-ATPase) rotary proton pumps. Oral toxicity towards insect pests and an absence of activity towards mammals makes Pa1b an attractive candidate for development as a bioinsecticide. The purpose of this study was to investigate if *Pichia pastoris* could be used to express a functional PA1b peptide and if it’s insecticidal activity could be enhanced via engineering to produce a fusion protein comprising the pea albumin protein fused to the mannose-specific snowdrop lectin (*Galanthus nivalis* agglutinin; GNA)*.*

**Results:**

We report the production of a recombinant full-length pea albumin protein (designated PAF) and a fusion protein (PAF/GNA) comprised of PAF fused to the N-terminus of GNA in the yeast *Pichia pastoris*. PAF was orally toxic to pea (*Acyrthosiphon pisum*) and peach potato (*Myzus persicae*) aphids with respective, Day 5 LC_50_ values of 54 µM and 105 µM derived from dose–response assays. PAF/GNA was significantly more orally toxic as compared to PAF, with LC_50_ values tenfold (5 µM) and 3.3-fold (32 µM) lower for pea and peach potato aphids, respectively. By contrast, no phenotypic effects were observed for worker bumble bees (*Bombus terristrus*) fed PAF, GNA or PAF/GNA in acute toxicity assays. Confocal microscopy of pea aphid guts after pulse-chase feeding fluorescently labelled proteins provides evidence that enhanced efficacy of the fusion protein is attributable to localisation and retention of PAF/GNA to the gut epithelium. In contact assays the fusion protein was also found to be significantly more toxic towards *A. pisum* as compared to PAF, GNA or a combination of the two proteins.

**Conclusions:**

Our results suggest that GNA mediated binding to V-type ATPase pumps acts to potentiate the oral and contact aphicidal activity of PAF. This work highlights potential for the future commercial development of plant protein-based bioinsecticides that offer enhanced target specificity as compared to chemical pesticides, and compatibility with integrated pest management strategies.

**Supplementary Information:**

The online version contains supplementary material available at 10.1186/s12934-023-02176-1.

## Background

It is now more than 35 years since John A. Gatehouse and collaborators, to whom this manuscript is dedicated, discovered a sulphur rich pea albumin protein (Psa LA, now designated PA1) in *Pisum sativum* seeds [1]. Whilst Gatehouse et al. [1] suggested that PA1 may play a role as an anti-metabolite against seed attacking pests, early functional studies did not support this hypothesis, and it was initially thought to be a novel storage protein. Studies in the late 1980s and early 1990s found PA1 to be expressed in seeds as a preproprotein (11 kDa) from which the signal peptide is cleaved in the endoplasmic reticulum. The protein is subsequently directed to a vacuole-like protein storage organelle where it is cleaved into its mature proteins, PA1a (6 kDa) and PA1b (4 kDa) [2]. The first report of insecticidal activity appeared in 1993 where 80–100% mortality of 3 out of 4 species of cereal weevil (*Sitophilus sp.*) fed on split peas was reported [3]. Insecticidal activity was eventually attributed to the PA1b peptide and its use as an insecticidal toxin, based upon toxicity to pea aphids *Acrythosiphon pisum* and Mediterranean flour moth (*Ephestia kuehniella*), was initially patented in 1998 [4].

Pa1b is a 37 amino acid peptide containing 6 cysteine residues that form 3 intramolecular disulfide bridges and is a member of the inhibitor cysteine knot (ICK) peptide family [5]. Evolutionarily conserved across phyla and functionally diverse, ICK peptides are particularly abundant in cone snail and spider venoms and known to inhibit voltage and ligand gated ion channels [6–8]. Based upon the structural similarity of PA1b to an ICK atracotoxin (ACTX-Hi:OB4219) present in the venom of the Australian funnel web spider *Hadronyche infensa*, Jouvensal et al. [9] hypothesised Pa1b may have a similar ion channel blocking function. Electrophysiological and pharmacological studies subsequently identified the target site of action of PA1b to be vacuolar adenosine triphosphatase (V-ATPase) rotary proton pumps [10]. Ubiquitous throughout eukaryotic membranes, V-ATPases are responsible for organelle acidification and membrane energisation and drive multiple secondary transport functions [11, 12]. Comprised of 14 polypeptides, the holoenzyme forms two complexes; a cytoplasmic facing ATP-hydrolytic domain V_1_ comprised of subunits A-H, and a membrane associated proton transport domain V_0,_ formed of several transmembrane subunits, a, d, e, c, and c’’ (reviewed by [13]). PA1b acts by binding and inhibiting subunits c and e of the V_0_ complex, both of which have exposure on the apical side of the gut membrane and are involved in the rotor function of V-ATPases [14, 15]. Pa1b binding and insecticidal activity are both dependent on a cluster of residues in L2, between the first and second β-sheets, with binding activity dependent on Phe-10, Arg-21, Ile-23 and Leu-27 residues [16].

PA1b is the only known proteinaceous inhibitor of V-ATPase proton pumps. Significant oral insecticidal activity against certain insects, notably cereal weevils (genus *Sitophilus*), mosquitoes *Culex pipiens* and *Aedes aegyptii*, and certain species of aphids, together with non-toxicity towards mammals or other non-insect organisms makes PA1b an interesting candidate for development as a bioinsecticide [17, 18]. The ICK motif is known to confer high proteolytic, thermal, and chemical stability, making it suitable for scaleable expression as a recombinant protein. Potential for the exploitation of ICK peptides as novel bioinsecticides is demonstrated by the product Spear^®^-T which contains a recombinant spider venom peptide GS-ω/κ-HxTx-Hv1h (named as GS-ω/κ-HxTx-Hv1a by Vestaron) as the active ingredient. Spear^®^-T is sold as a contact foliar spray for the control of glasshouse pests and the venom peptide is produced using a yeast based fermentation system (vestaron.com).

We have previously reported that *Pichia pastoris* is able to produce functional recombinant ICK peptides (where di-sulphide bridge formation is essential for peptide activity), and that fusion of spider venom derived ICK peptides SFI1, Hv1a, and HxTx-Hv1h to snowdrop lectin (*Galanthus nivalis* agglutinin; GNA) significantly enhances oral and contact activity towards a range of insect pests [19–23]. ICK insecticidal activity is enhanced by virtue of the ability of GNA to transport attached venom peptides across the gut epithelium into the circulatory system, thereby enabling attached venom toxins to reach their target sites of action in the central nervous system. We have also reported the ability of GNA to facilitate retention of fused toxin to the aphid gut via binding to the gut epithelium [21, 23]. In this study we hypothesised that fusion to GNA may result in enhanced toxicity of the pea albumin peptide by virtue of the ability of the mannose-specific lectin to bind to epithelial V-ATPase pumps, which are known to be heavily glycosylated, and thereby localise or “deliver” Pa1b to its target site of action [24].

Heterologous expression of the PA1b peptide alone has proved problematic posing challenges for the development of large-scale microbial production systems to produce protein-based bioinsecticides. Eyraud et al. [5] reported unsuccessful attempts to express the PA1b peptide via transient expression in tobacco (*Nicotiana benthamiana*) and that recombinant production of PA1b in bacteria (*E. coli*) or yeast (*P. pastoris*) had never been successful. By contrast, expression of full length PA1 in tobacco was successful, indicating possible roles for PA1a in RNA stabilisation and/or as a chaperone for the correct folding of the PA1b peptide [5]. Our initial attempts to express the PA1b peptide in *P. pastoris* also proved challenging due to relatively low levels of expression (ca. 5 mg/L culture supernatant) and difficulties in purifying the peptide from fermented cultures. Here we report the production of full length PA1 (comprised of the PA1a and Pa1b peptides) and PA1 fused to the N-terminus of GNA in the yeast *P. pastoris*. Oral and contact activity of PA1 and PA1/GNA towards aphids is reported alongside fluorescence microscopy of whole guts extracted from pea aphids chase-fed on labelled proteins. The potential for off-target effects against bumblebees (*Bombus terrestris*) has also been investigated.

## Results

### Recombinant protein production in the yeast *P. pastoris*

**Fig. 1:**
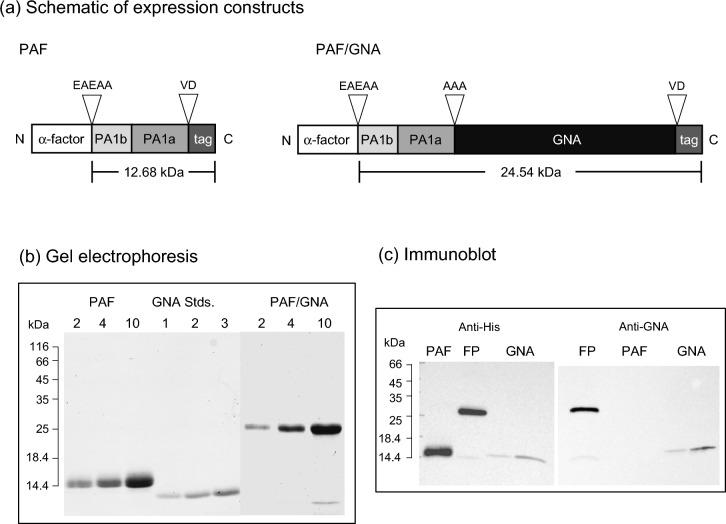
**a** Schematic of constructs encoding recombinant PAF and PAF/GNA produced in the yeast *P. pastoris* showing predicted molecular masses; tag denotes the presence of a six-residue histidine sequence enabling protein purification by nickel affinity chromatography and immunoblot detection. **b** Separation of purified proteins by SDS-PAGE gel (17.5% acrylamide) stained for total protein: loading of proteins in µg is depicted. GNA standards are GNA purified from snowdrop bulbs (Sigma-Aldrich, St. Louis, USA). **c** Western analysis of recombinant proteins using anti-His (400 ng protein loaded) and anti-GNA (200 ng protein loaded) antibodies. Location of mass markers run on the same gel are depicted

Synthetic genes encoding PA1 without the signal peptide (NCBI Accession P62930 residues 33–103), hereafter referred to as pea albumin full (PAF), and PAF/GNA were cloned in frame with the yeast alpha factor in the expression vector pGAPZαB by PCR amplification, followed by restriction, and ligation. The full length PAF protein was fused to the N-terminus of GNA via a three amino acid residue, (Ala-Ala- Ala) linker region as depicted in Fig. 1a. Both expression constructs contain a C-terminal six-residue histidine tag to enable immunoblot detection and affinity purification. Constructs were cloned into *E. coli* and sequenced plasmid DNAs were linearised and transformed into competent *P. pastoris* cells. Small scale screening by western blotting for protein expression enabled the selection of clones for bench-top fermentation to produce sufficient protein for insect bioassays. *P. pastoris* cells were grown in a bench-top fermenter and recombinant proteins were purified from clarified supernatants by nickel-affinity chromatography, followed by dialysis and freeze drying. PAF and PAF/GNA were expressed at respective levels of ca. 40 and 80 mg/L culture supernatant.

As shown in Fig. 1b, purified recombinant PAF separates as two protein products of approximately 14 kDa on SDS-PAGE gels, which is close to the predicted mass of 12.7 kDa. Immunoreactivity with anti-His antibodies (Fig. 1c) provides evidence that both proteins represent recombinant PAF. Staining by periodic acid Schiff blot analysis confirmed glycosylation of recombinant PAF (but not PAF/GNA) and thus differential glycosylation may account for the presence of two protein products (Additional File 1). Liquid chromatography–mass spectrometry (LC–MS) analysis of PAF confirmed that the proteins are full length, comprised of both the PA1b and PA1a peptides, have identical sequence, and contain additional N-terminal residues (Glu-Ala-Glu-Ala-Ala) due to incomplete processing of the alpha factor sequence by yeast dipeptidyl aminopeptidase (Additional Files 2 and 3). Additional N-terminal alanine and C-terminal valine and aspartic acid residues are derived from gene insertion via *Pst I* and *Sal I* restriction sites. Purified PAF/GNA separates as a single protein of approx. 25 kDa on SDS-PAGE gels, close to the 24.5 kDa predicted mass (Fig. 1a, b) and a minor product of ca. 14 kDa. Both proteins reacted positively with anti-GNA and anti-His antibodies suggesting that the 25 kDa product is intact PAF/GNA and the minor 14 kDa protein is cleaved GNA (Fig. 1c). LC–MS analysis of the 25 kDa protein confirmed the presence of full-length sequence and, as for PAF, the presence of additional N-terminal and C-terminal residues (Additional File 2, 3). As previously reported recombinant GNA which contains a C-terminal histidine tag separates at approximately 14 kDa on SDS-PAGE gels (Fig. 1b, c), close to its predicted molecular mass of 12.8 kDa [22].

### Biological activity of recombinant proteins

#### Oral toxicity of recombinant proteins to aphids

**Fig. 2:**
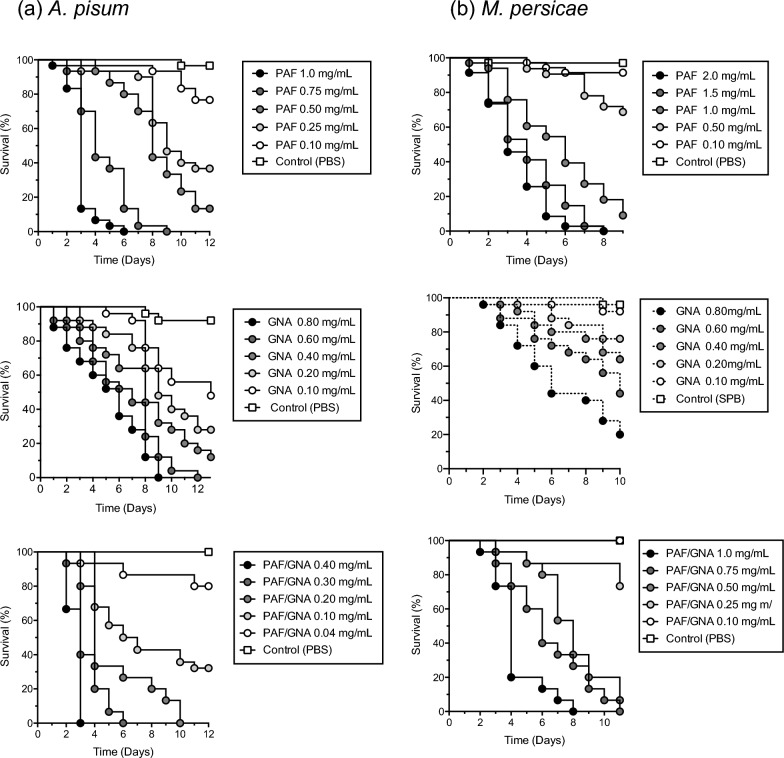
Survival of (**a**) *A. pisum* and (**b**) *M. persicae* fed on artificial diets containing different concentrations of purified recombinant PAF, GNA or PAF/GNA

Oral toxicity was determined by feeding *A. pisum* or *M. persicae* nymphs with artificial diets containing a range of concentrations (0.04–2.0 mg/mL) of recombinant PAF, GNA, or PAF/GNA. As shown in Fig. 2 dose dependent reductions in the survival of aphids fed on protein containing diets were observed in all assays, whereas control (no added protein diet) survival was > 90%. For PAF/GNA preliminary tests indicated that the fusion protein had to be fed at lower concentrations, as compared to PAF or GNA, to allow LC_50_ values to be calculated. Mortality reached 100% for pea aphids (*A. pisum*) fed on the highest concentrations of PAF, GNA or PAF/GNA. All protein treatment curves were significantly different to control survival (Kaplan–Meier survival analysis; P < 0.05). Mortality also reached 100% for peach potato aphids (*M. persicae*) fed on high doses of PAF or PAF/GNA (> 0.75 mg/mL), but only reached 80% for GNA at the highest concentration of 0.8 mg/mL. All treatment curves for *M. persicae*, except for PAF/GNA at 0.1 mg/mL, were significantly different to control survival (Kaplan–Meier survival analysis; P < 0.05). As shown in Table 1, the derived day 5 LC_50_ value of 54 µM for pea aphids fed on PAF containing diets is almost twofold lower as compared peach potato aphids indicating that PAF is considerably more toxic to *A. pisum* as compared to *M. persicae*; this difference in efficacy was also observed for GNA although to a lesser degree (62 µM for *A. pisum* as compared to 78 µM for *M. persicae*). The fusion protein was found to be considerably more orally toxic to both aphid species as compared to either PAF or GNA alone. LC_50_ values for PAF/GNA were more than tenfold lower for *A. pisum* and 3.3-fold lower for *M. persicae* as compared to PAF alone and respectively, 12.4-fold and 2.4-fold lower as compared to GNA.

**Table 1:** Day 5 LC_50_ values (mg/mL and µM) derived from bioassay data in Fig. 2a and b

Protein	Pea aphid (*A. pisum*)				Peach-potato aphid (*M. persicae*)			
	mg/mL		µM		mg/mL		µM	
	LC_50_	95% C.I	LC_50_	95% C.I	LC_50_	95% C.I	LC_50_	95% C.I
PAF	0.68	0.62–0.75	54	49–60	1.33	1.32–1.36	105	104–107
GNA	0.80	0.64–0.99	62	49–77	1.01	0.83–1.21	78	65–94
PAF/GNA	0.12	0.09–0.17	5	4–7	0.79	0.69–0.90	32	28–37

C.I. depicts confidence intervals

#### Retention of PAF and PAF/GNA in the *A. pisum* gut

**Fig. 3:**
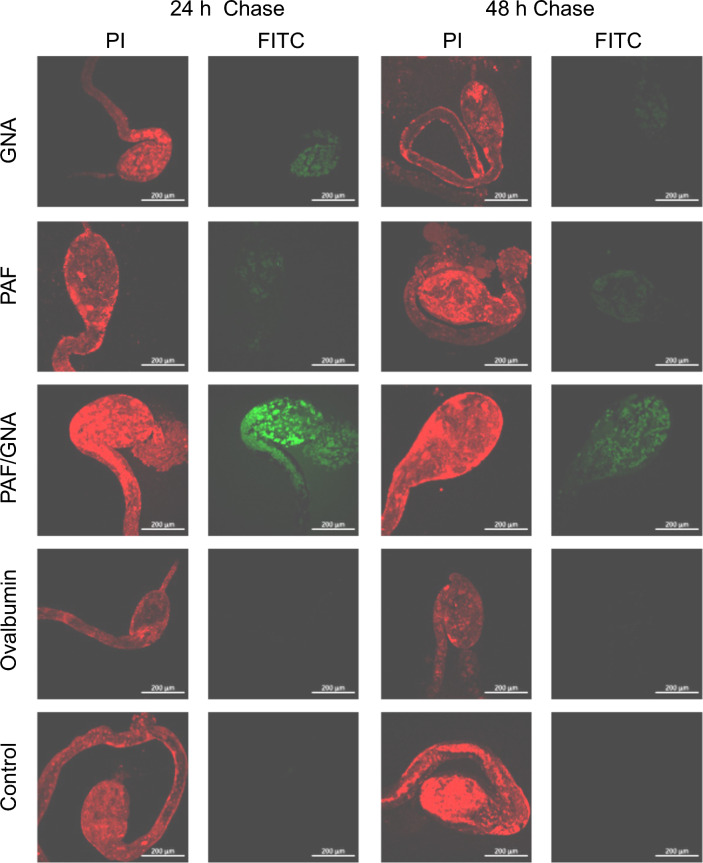
Representative confocal images of *A. pisum* dissected guts in a pulse-chase feeding experiment with FITC labelled proteins. Five-day-old aphids were fed for 24 h on artificial diets containing FITC labelled proteins (8 µM of either PAF, GNA, or PAF/GNA or 5 µM ovalbumin), or control (no protein) diet and counterstain PI followed by chase feeding on artificial diet plus PI for 24 or 48 h. Guts were dissected in sterile ice-cold PBS and imaged immediately using a Zeiss 880 LSM

**Fig. 4:**
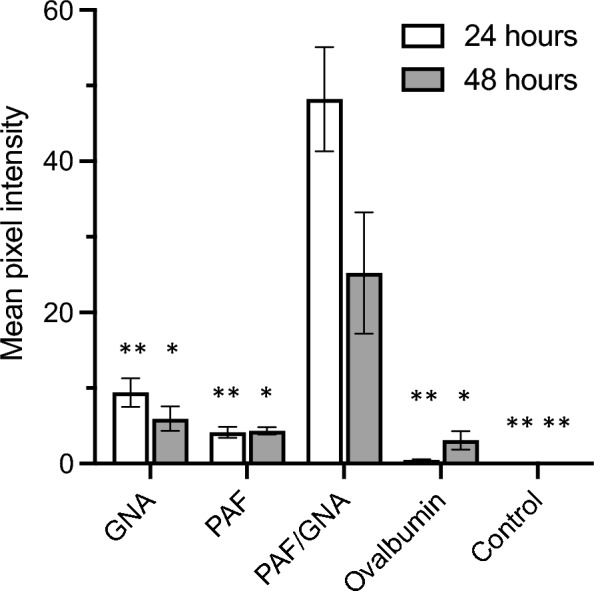
Average pixel intensity of *A. pisum* guts from confocal imaging of a pulse-chase experiment with FITC labelled proteins (representative images in Fig. 3). GNA, PAF and PAF/GNA were fed at 8 µM and ovalbumin at 5 µM*.* Pixel intensity was measured using the measure function of FIJI, the gut was traced using the outline provided by the PI counterstain and fluorescence in the green wavelength recorded. Mean pixel intensity was recorded for a minimum of 6 guts per treatment and standard error was calculated. Pairwise t-tests were used to determine statistical significance * denotes p < 0.05 or ** p < 0.0005 when compared to equivalent PAF/GNA timepoint

To investigate if differences existed in the persistence and/or binding of the proteins to the aphid gut epithelia a pulse-chase experiment was conducted. Pea aphids were fed on equimolar concentrations of FITC labelled proteins for 24 h and subsequently chase fed (for 24 and 48 h) on propidium iodide (PI) counterstain only diets. Ovalbumin was used as a control as it should not interact with the epithelial membrane. Representative fluorescence imagery of dissected gut samples is presented in Fig. 3. Fluorescence was not detected in guts derived from FITC-ovalbumin fed aphids but was detected in PAF, GNA, and PAF/GNA treatments after chase feeding for 24 h and reduced fluorescence is visible in guts extracted after 48 h. In initial experiments PAF and GNA displayed intense fluorescence when imaged after 24 and 48 h. However, due to the increased fluorescence observed in the PAF/GNA treatments, laser power had to be reduced to prevent saturation of the PAF/GNA images and consistency across conditions was required to allow accurate measurement of pixel intensity across all images using standardised imaging settings. In all cases, where visible, the proteins are localised to the bulbous stomach region although staining of the abdominal loop is also visible. The intensity of fluorescence was notably higher in guts dissected from PAF/GNA fed aphids after both 24 and 48 h of chase feeding as compared to PAF or GNA. This pattern was also observed when the mean pixel intensity was quantified and averaged across multiple dissected samples. As shown in Fig. 4, mean pixel intensity was significantly greater for PAF/GNA as compared to all the other conditions tested across both time points (24 h: p < 0.0005; 48 h: p < 0.05; t-test).

#### Contact toxicity *A. pisum*

**Fig. 5:**
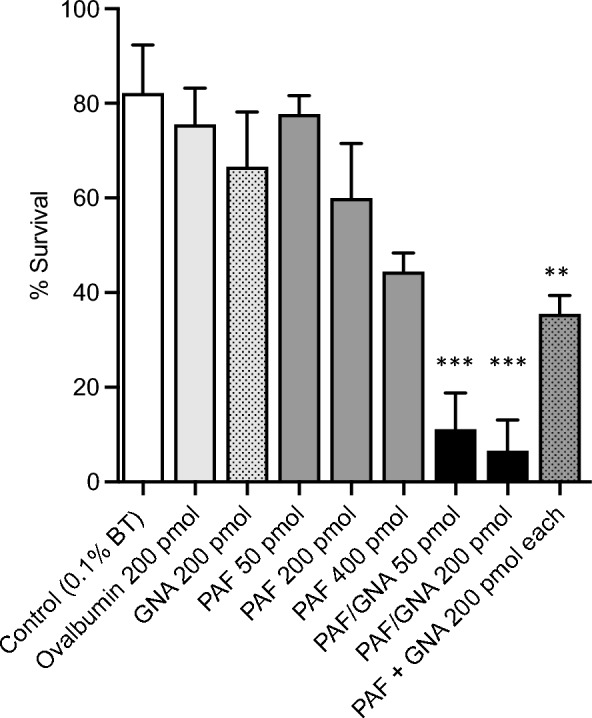
Pea aphid survival 24 h after contact exposure to water + Breakthru (BT), ovalbumin, GNA, PAF, PAF/GNA or a mixture of GNA and PAF. All protein treatments contained BT. Bars depict standard error of the mean (3 replicates of n = 15 per dose), * denotes a significant difference to control (+ BT) and ovalbumin treatments at ** P < 0.005, and ***P < 0.0005 (t-tests)

The efficacy of topically applied proteins was evaluated by placing pea aphids in ventral contact with droplets containing different concentrations of ovalbumin (control), PAF, PAF/GNA or GNA and a mixture of PAF and GNA. As shown in Fig. 5, the mean survival of aphids 24 h after exposure to Break-thru alone (BT control) was more than 80%, and 75% for the ovalbumin control protein treatment. Survival following exposure to GNA or lower doses of PAF (50 and 200 pmol) was lower than the control treatments but not significantly so. Exposure to the highest PAF concentration (400 pmol) caused a significant decline in survival as compared to the control (P < 0.005; t-test) as did the combination treatment (200 pmol of GNA and 200 pmol PAF; P < 0.005; t-test); this suggests PAF does have contact activity, albeit at high doses, and that the effects of GNA and PAF are additive. By contrast, significant differences in survival as compared to the BT control were observed for both PAF/GNA treatments (50 and 200 pmol; respectively, P < 0.005 and P < 0.0005; t-tests). That the fusion protein is significantly more toxic as compared to PAF alone is shown by significant differences between the survival of PAF/GNA and PAF treated aphids observed for both 50 and 200 pmol treatments (P < 0.005; t-tests). That efficacy is enhanced as a result of fusion of PAF to GNA, rather than additive effects of the individual components, is evidenced by the significantly greater mortality of aphids exposed to 200 pmol of PAF/GNA as compared to an equivalent mixture of 200 pmol each of PAF and GNA (P < 0.005; t-test). These results provide evidence that fusion of PAF to GNA significantly enhances contact efficacy of the pea albumin protein towards *A. pisum*.

#### Bumblee (*B. terristris*) assays

To test the potential for negative effects of PAF/GNA to pollinators an acute oral toxicity assay where bumble bee workers were fed on sucrose solutions containing recombinant proteins was conducted. The positive control, Dimethoate caused 100% mortality 24 h after each individual bee had consumed a total of 4 μg product. By contrast, 100% survival was observed over an assay period of 7 days for bees exposed to PAF (426 μg/bee), GNA (323 μg/bee) or PAF/GNA (257 μg/bee). No phenotypic effects were observed suggesting that all proteins were effectively non-toxic to adult bumble bees.

## Discussion

The pea seed PA1b ICK peptide has oral toxicity towards a range of insect pests through selective inhibition of V-ATPase proton pumps that offers potential for it’s exploitation as a bioinsecticide [3, 4, 15, 17, 25]. The purpose of this study was to investigate if PA1b could be produced recombinantly using the yeast *P. pastoris*, and secondly to elucidate if fusion to snowdrop lectin, GNA would potentiate the insecticidal activity of PA1b towards aphid pests.

To date, reports of heterologous expression of pea albumin have largely been limited to *in planta* studies where the full-length PA1 protein has been shown to be expressed and processed correctly in white clover (*Trifolium repens*) [26]. Eyraud et al. [5] further confirmed that a full-length PA1 cassette, rather than the PA1b peptide alone, was necessary for transient expression of the PA1b peptide in tobacco (*N. benthiamina*). Jackson et al. [27] also used a full-length PA1 construct to express PA1b in a CRISPR edited *N. benthiamina* line. We similarly found expression of the PA1b peptide in yeast to be problematic providing further support for a role for PA1a in ensuring the correct folding of the PA1b peptide. Whereas PA1 expression *in planta* results in processed forms of Pa1b, immunoblot and LCMS analysis confirmed that PA1 (PAF herein) is expressed as full-length protein in yeast with both PA1a and PA1b peptides present in the final protein products. Respective PAF and PAF/GNA expression levels of 40 and 80 mg/L yeast culture supernatant were obtained. Purified recombinant proteins were used to evaluate efficacy towards two pest aphid species (*A. pisum* and *M. persicae*) and adult bumble bees (*B. terrestris*) as a non-target pollinator species.

In feeding assays PAF was found to be chronically toxic to pea (*A. pisum*) and, to a lesser extent, peach potato (*M. persicae*) aphids, with respective derived day 5 LC_50_ values of 54 µM (0.68 mg/mL) and 105 µM (1.33 mg/mL). For pea aphids 100% mortality was observed after 6 days of feeding on a diet containing 1 mg/mL (79 µM). These data are comparable to Gressent et al. [25] who reported 57% *A. pisum* mortality after feeding on PA1b (purified from pea seeds) at 0.15 mg/mL (ca. 40 µM based upon a peptide mass of 3.68 kDa) and 100% mortality at 0.5 mg/mL (135 µM). Surprisingly, Gressent et al., [25] reported no effect upon *M. persicae* survival at concentrations of up to 1.0 mg/mL (270 µM) whereas we observed 100% mortality after 6 days feeding on 2.0 mg/mL (159 µM) PAF. This suggests that the full-length recombinant PAF was similarly toxic to PA1b derived from pea seeds and that glycosylation of protein did not alter its insecticidal properties. Gressent et al. [25] hypothesised that differences in Pa1b toxicity towards different insect species may be attributable to variations in binding affinity and/or stability to proteolysis. However, no correlation between either factors with efficacy was observed; Kd values for *A. pisum* and *M. persicae* were similar (58 and 62 nM, respectively) and the peptide was shown to be resistant to enzymatic degradation. Muench et al. [15] hypothesised that the insect selectivity of PA1b may be attributable to sequence variability in the extreme N and C-termini and extracellular loop of sub-unit c. This hypothesis is not supported by the observed difference in PAF efficacy together with the 100% identity of *A. pisum* and *M. persicae* amino acid sequences for PA1b binding sites V-type ATPase sub-units c (respective Accession Nos. NP_001155531.1; XP_022173722.1) and e (respective Accession Nos XP_003242132.1 and XP_022172967.1). Our attempts to assess the stability of PAF to proteolysis by western blotting of aphid extracts were unsuccessful due to cleavage of the histidine tag present in PAF. As such, the reason for the observed differences in PAF efficacy against the two aphid species remain to be determined.

GNA itself was also chronically toxic towards *A. pisum* and *M. persicae*. As for PAF, GNA was more effective against pea aphids (day 5 LC_50_ 0.80 mg/mL; 62 µM) as compared to peach potato aphids (day 5 LC_50_ 1.01 mg/mL; 78 µM). The insecticidal effects of mannose binding lectins, such as GNA towards homopteran pests is well documented, and our results are comparable to previous artificial diet and transgenic studies that show GNA to be toxic to aphid pests [23, 28–30].

Recombinant PAF/GNA was significantly more orally toxic than PAF or GNA alone towards both aphid species. Derived LC_50_ values for *A. pisum* and *M. persicae* were respectively, ca. tenfold lower and 3.3-fold lower for PAF/GNA, as compared to PAF or GNA alone. It is possible that the enhanced oral activity of the fusion protein may be due to the synergistic action of PAF and GNA although we have previously observed only additive effects in aphid assays where we have fed a mixture of an insecticidal recombinant ICK peptide together with GNA [23]). That the enhanced activity of PAF/GNA is attributable to fusion of PAF to GNA rather than synergistic oral activity of the two proteins is supported by confocal microscopy of pea aphid guts dissected after pulse-chase feeding of fluorescently labelled proteins. Significantly greater fluorescence intensity of guts dissected from PAF/GNA fed aphids as compared to those from PAF or GNA fed aphids provided evidence that the significantly greater oral efficacy of the fusion protein is attributable to GNA-mediated enhanced localisation and retention of PAF/GNA to the gut epithelium. Previous studies have similarly provided evidence for GNA binding and retention within the posterior (stomach) region of hemipteran gut epithelia [21, 23, 31]. This region is associated with secretion and digestion and has been shown to be slightly more acidic than the remainder of the aphid gut which may imply an increased abundance of V-ATPase proton pumps [32, 33]. The V-ATPase Vo subunit (the target of PA1b) isolated from midgut and malpighian tubules of the tobacco hornworm, *Manduca sexta*, has been shown by mannose binding lectin Concanavalin A staining to be heavily glycosylated [24]. It is therefore possible that GNA may also bind to V-type ATPases in the midguts of *A. pisum* and *M. persicae* and thereby potentiate PAF induced inhibition of the rotor function of the pumps.

When applied topically PAF exhibited a low level of toxicity towards *A. pisum*. V-type ATPases are abundant throughout nearly all epithelial tissues of insects, regulating multiple processes including receptor-mediated endocytosis, protein degradation, fluid secretion, and neurotransmission [34, 35]. As such, by contacting internal epithelial tissues PAF may act to inhibit the many processes mediated by proton pumps, such as those present in salivary or neuronal epithelia. Contact toxicity of PAF towards pea aphids was significantly enhanced when fused to GNA. As only additive effects upon mortality were observed when aphids were exposed to a combination of GNA and PAF our results indicate, as for oral activity, that this may be mediated by GNA binding and delivery of PAF to its target site of action. Mannosylated proteins are abundant within insects [36] providing multiple binding possibilities for GNA, including the V_o_ sub-unit of V-type ATPases [24]. Pa1b disrupts V-type ATPase pumps via binding to apical membranes and this suggests that GNA must not only bind but also deliver Pa1b across basal epithelia to enable access of the peptide to the apically located Vo complex. It is well established that GNA binds to the apical gut epithelia, and transports fused peptides across to the haemolymph [19–21, 37]. However, GNA has also been shown to bind to the basal epithelia of *Spodoptera littoralis* midgut cells [38]. It is thus plausible that following topically mediated internalisation of the fusion protein that GNA “delivers” fused PAF to its target site of action via binding to basal epithelia and subsequent transport across to the apical membrane where the Vo complex is situated. Alternatively, GNA and/or Pa1b binding sites may be accessible on both the basal and apical surfaces of epithelia.

We have demonstrated that fusion of PAF to GNA potentiates the oral and contact activity of the pea albumin ICK Pa1b peptide towards two aphid species. We observed no toxic effects upon adult *B. terrestris* fed on > 200 µg fusion protein per bee. PA1b was previously reported to be orally toxic towards invasive Asian harlequin ladybeetles (*Harmonia axyridis*), causing significant levels of mortality when fed at 0.25 mg/mL over a period of 12 days [25]. Further studies are required to investigate the potential for detrimental effects upon beneficial pollinator and biocontrol insect species. We have previously shown that spider venom derived ICK peptides (HxTx-Hv1h and PI1a) known to target insect neuronal ion channels, are also significantly more effective against aphid species when fused to GNA [21, 23]. Respective Day 2 LC_50_ values of 35 µM and 33 µM for HxTx-Hv1h/GNA towards pea and peach potato aphids are broadly comparable to Day 5 LC_50_ values of 5 µM and 32 µM reported herein [23]. Contact toxicity towards pea aphids was also comparable to that previously reported for HxTx-Hv1h/GNA where 82% and 70% mortality was observed 24 h after exposure to 280 and 70 pmol protein, respectively. Collectively our data supports considerable potential for the use of the only proteinaceous inhibitor of insect V-type ATPase proton pumps Pa1b, when fused to GNA, as a novel biopesticide to control aphid pests.

## Conclusions

This is the first report of the successful expression of Pa1b using yeast as a host to express a full-length PA1 protein. We have produced, purified, and characterised a unique recombinant fusion protein that includes the pea seed knottin peptide (PA1b), that specifically targets insect V-type ATPase proton pumps, fused to the mannose-specific lectin GNA. Comparative aphid bioassays have demonstrated that the fusion protein is significantly more toxic, both orally and following contact exposure, than the individual protein components. Chase feed aphid assay confocal microscopy data indicates that enhanced aphicidal activity of the fusion protein is attributable to GNA-mediated binding/localisation of PA1b to V-type ATPase pumps. That this fusion protein is likely to have minimal impact upon pollinator species has also been indicated by an absence of phenotypic effects in bumble bees following acute oral exposure. These results highlight potential for the development of fermentation based approaches to produce protein-based bioinsecticides with modes and target sites of action that are distinct to the ion and ligand-gated ion channels that are commonly targeted by chemical pesticides.

## Methods

### Materials

A *P. pastoris* codon optimised nucleotide sequence (https://eu.idtdna.com/CodonOpt) encoding PA1 without the signal peptide (NCBI Accession P62930 residues 33–103), referred to as pea albumin full (PAF), and cloning primers were purchased from Integrated DNA Technologies (IDT). Restriction endonucleases were supplied by Thermo scientific or New England BioLabs. Electrophoresed DNA fragments were purified from excised gel slices using a Qiagen gel extraction kit. Plasmid DNA was prepared using Promega Wizard miniprep kits. T4 ligase kit was supplied by Promega. Phusion polymerase was from New England Biolabs. *Pichia pastoris* (SMD1168H strain), the expression vector pGAPZαB, and Easy comp *Pichia* transformation kit were from Invitrogen.

Anti-GNA antibodies were prepared by Genosys Biotechnologies, Cambridge, UK. Monoclonal 6x-His Tag Antibodies were from Fisher Scientific, UK. Secondary IgG horseradish peroxidase antibodies were from Biorad. Chemicals for chemiluminescence and buffer salts were supplied by Sigma.

### Assembly of PAF and PAF/GNA fusion protein expression constructs

The PAF coding sequence was amplified by PCR using primers containing *Pst*I and *Sal*I restriction sites. Following gel purification, the PCR product was digested (*Pst*I and *Sal*I) and ligated into similarly cut vector pGAPZαB DNA. To generate a fusion protein expression construct where PAF is linked to the N-terminus of GNA, the pea albumin coding sequence was amplified by PCR (using primers containing *Pst*I and *Not*I restriction sites), gel purified, restricted, and ligated into a previously generated pGAPZαB construct that contained a GNA coding sequence. Plasmids were cloned into electrocompetent *E. coli* (DH5α) cells and DNA coding sequences were verified by “in house” DNA sequencing and analysed using Serial Cloner 2.6.

### Yeast transformation, expression, and purification of recombinant proteins

DNAs from sequence verified clones were linearised with *Avr*II and transformed into chemically competent *P. pastoris* cells according to the manufacturer’s instructions. Transformants were selected on media containing 100 μg/mL zeocin. Clones expressing recombinant PAF or PAF/GNA were selected for production by bench-top fermentation by Western analysis (using anti-His or anti-GNA antibodies) of supernatants from 10 mL cultures grown at 30° C for 2–3 days in YPG medium (1% [w/v] yeast extract, 2% [w/v] peptone, 4% [v/v] glycerol, 100 ug/mL zeocin).

For protein production *P. pastoris* cells expressing PAF or PAF/GNA or GNA were grown in a bench-top fermenter (ez-control Applikon 7.5 L vessel) as previously described [20]. Following fermentation, proteins were separated from cells by centrifugation (20 min at 7000 *g*, 4 °C) and purified via nickel affinity chromatography as previously described [22]. Pooled fractions containing purified proteins were dialysed against dist. water and lyophilised. Protein contents in lyophilised samples were determined from SDS-PAGE gels stained for total proteins with Coomassie blue. Quantitation was based on bands corresponding to intact proteins, which were compared to GNA (Sigma) standards by visual inspection, and iBright analysis of gel images scanned using a commercial flat-bed scanner.

### Electrophoresis, Western blotting and Fluorescein conjugation

SDS-PAGE electrophoresis, western blotting, and fluorescein isothiocyanate (FITC) labelling of recombinant proteins was carried out as described previously [20].

### Recombinant protein characterization

Recombinant PAF and PAF/GNA were separated by SDS-PAGE and excised bands from gels stained with Coomassie Blue were analysed by LC–MS. Proteins in excised bands were digested with chymotrypsin or ProAlanase, and LC–MS analysis was performed with a Sciex TripleTOF 6600 mass spectrometer coupled to an ekspertTM nanoLC 425 with low micro-gradient flow module (Eksigent) via a DuoSpray source (Sciex) as described previously [22].

### Insect rearing

*Acyrthosiphon pisum* (pea aphid) and *Myzus persicae* (peach potato aphid) were reared on broad bean (*Vicia faba*) and Chinese cabbage (*Brassica rapa*), respectively, and both colonies were maintained at 22 °C with a 16 h light: 8 h dark cycle.

### Aphid feeding assays

Oral toxicity to *A. pisum* and *M. persicae* was determined using cylindrical feeding chambers overlain with parafilm sandwiches that contained proteins dissolved in liquid artificial diet [39]. Stock proteins solutions in phosphate buffered saline (PBS; 10 × stock 0.015 M KH_2_PO_4_, 0.08 M Na_2_HPO_4_, 1.37 M NaCl in dist. water pH 7.4) were added to sterile diet such that 100 μL diet contained 25 μL protein solution. Control diets contained an equivalent volume of PBS to the protein treatments. One-day-old nymphs, selected from adults maintained on artificial diet for 24–48 h, were placed on 25 μL artificial diet (15 nymphs per dose). Diets were replaced every 2 days and survival recorded daily. Preliminary assays enabled determination of appropriate protein concentrations to allow derivation of median lethal (LC_50_) concentrations.

### Fluorescence microscopy

Five-day-old pea aphids were fed for 24 h on artificial diets containing 8 µM of either PAF/GNA, PAF, GNA, or ovalbumin, or control (no protein) diet with PI counterstain (100 ng/µL) and subsequently chased with artificial diet and PI for 24 or 48 h. Aphid guts were dissected by submerging live adults in ice-cold PBS and fine tip forceps were used to gently separate the gut from the head and the body. Dissected guts were mounted directly onto glass slides in Vectashield H-100, overlaid with glass coverslips, and visualised using a Zeiss 880 confocal laser scanning microscope. Settings were as follows: Objective Plan-Apochormat 20x/0.8 M27; FITC excitation and image capture: 488 nm laser (0.5% power), wavelengths collected between 493 and 572 nm, gain 575; PI excitation and image capture: 543 nm laser (1.5% power), wavelengths collected between 584 and 735 nm, gain 800. To quantify the fluorescence in each treatment a minimum of 6 guts from each condition were imaged utilising the same laser and image settings. The outline of the PI counterstain was traced in FIJI, an ImageJ based analysis software and the intensity of the green channel measured using the pixel intensity measuring tool in the base software package [40, 41]. The mean intensity of the traced area was then averaged, and the standard error calculated.

### Aphid (*A. pisum*) topical assays

The impact of contact exposure to recombinant PAF/GNA, PAF, GNA, or ovalbumin (as a control protein) was investigated using methods described previously [23]. In brief, anaesthetised aphids were individually placed in ventral contact with a 0.5 μL droplet of protein solution (in 0.1% [v/v] agricultural adjuvant Breakthru) and then placed in feeding chambers. Three biological replicates (15 aphids per replicate) were conducted for each protein treatment and dose; survival was recorded 24 h post treatment.

### Bumblebee (*B. terrestris*) assays

Acute oral toxicity towards adult bumble bees (*Bombus terrestris*) was investigated to assess the potential for off-target effects of PAF/GNA, PAF, or GNA. Methods were based upon OECD guidelines [42]. Total protein doses for the assays were 4 × the LC_50_ values derived from *M. persicae* feeding assays (i.e. the more robust of the two species to the toxic effects of PAF, GNA and PAF/GNA). Worker bees (150 mg average wt.) were maintained at 25 °C, 60% RH for 2–3 h, briefly anaesthetised, and transferred to individual nicot cages (queen rearing devices) and provided with a 50% (w/v) sucrose solution for approx. 16 h. Following acclimatisation, the bees (10 per treatment) were starved for 4 h to encourage feeding and then each provided with 80 µL of sucrose solution containing PAF (426 μg/bee), GNA (323 μg/bee), PAF/GNA (257 μg/bee) or Dimethoate (4 μg/bee) as a positive control, and sucrose only as a negative control treatment. All diet was consumed during the 4 h treatment period, after which the bees were fed on sucrose solution ad libitum*.* Mortality and phenotypic effects were monitored for a period of 7 days.

### Statistical analysis

Statistical analysis was carried out using GraphPad Prism or Microsoft Excel software. Survival data were analysed using Kaplan–Meier survival analysis. Median lethal doses or concentrations were calculated by plotting log transformed data and non-linear regression, constrained for control survival where necessary. Multiple t-tests were performed on contact assays for significant differences between single values. For fluorescence images Unpaired T-tests with Welch’s correction were performed to determine significant differences in mean intensity of traced areas as compared to the PAF/GNA condition.

### Supplementary Information

**Additional file 1: **Schiff blot to detect glycosylation status of recombinant PAF and PAF/GNA from fermented yeast cultures. Description: (a) Schiff Blot 10 µg protein loaded in each lane. (b) Coomassie Stained SDS-PAGE gel, 5 µg loaded in each lane. A, B, and C depict samples from different fermentations. FP6 denotes positive control recombinant fusion protein PI1a/GNA^21^. GNA: Commercially purified GNA (Sigma-Aldrich). Position of protein marker mix run on the same gel is depicted on the left-hand side.

**Additional file 2: **Primary structure of recombinant proteins - LC MS data Description: a) Primary structure of recombinant proteins expressed by transformed *P. pastoris *cells. Additional residues EAEAAA remain in expressed products due to incomplete processing of the alpha factor sequence by yeast dipeptidyl aminopeptidase, and the additional alanine is a consequence of gene insertion via a *Pst I *restriction site. The PA1b sequence is depicted in red, PA1a in blue, GNA in green and the linker region and histidine tags are in black. Remaining residues are the result of the cloning process for the expression construct. (b) LC-MS data obtained from ProAlanase and chymotrypsin digests of the PAF and PAF/GNA protein products. Blue bars depict identified peptides.

**Additional file 3: **Examples of LC-MS spectra obtained following fragmentation of PAF and PAF/GNA proteins. Description: LC-MS spectra.

## Data Availability

The datasets used and analysed during the current study are available from the corresponding author on reasonable request.
