# Supplementary material for: Heterologous production of the insecticidal pea seed albumin PA1 protein by Pichia pastoris and protein engineering to potentiate aphicidal activity via fusion to snowdrop lectin Galanthus nivalis agglutinin; GNA)
Source: Microb Cell Fact. 2023 Aug 17;22:157. doi: 10.1186/s12934-023-02176-1 (PMC10436433; doi:10.1186/s12934-023-02176-1)
Supplement: Supplementary file 1 — Additional file 1: Schiff blot to detect glycosylation status of recombinant PAF and PAF/GNA from fermented yeast cultures. Description: (a) Schiff Blot 10 µg protein loaded in each lane. (b) Coomassie Stained SDS-PAGE gel, 5 µg loaded in each lane. A, B, and C depict samples from different fermentations. FP6 denotes positive control recombinant fusion protein PI1a/GNA21. GNA: Commercially purified GNA (Sigma-Aldrich). Position of protein marker mix run on the same gel is depicted on the left-hand side. [file 12934_2023_2176_MOESM1_ESM.pptx]

## Slide 1
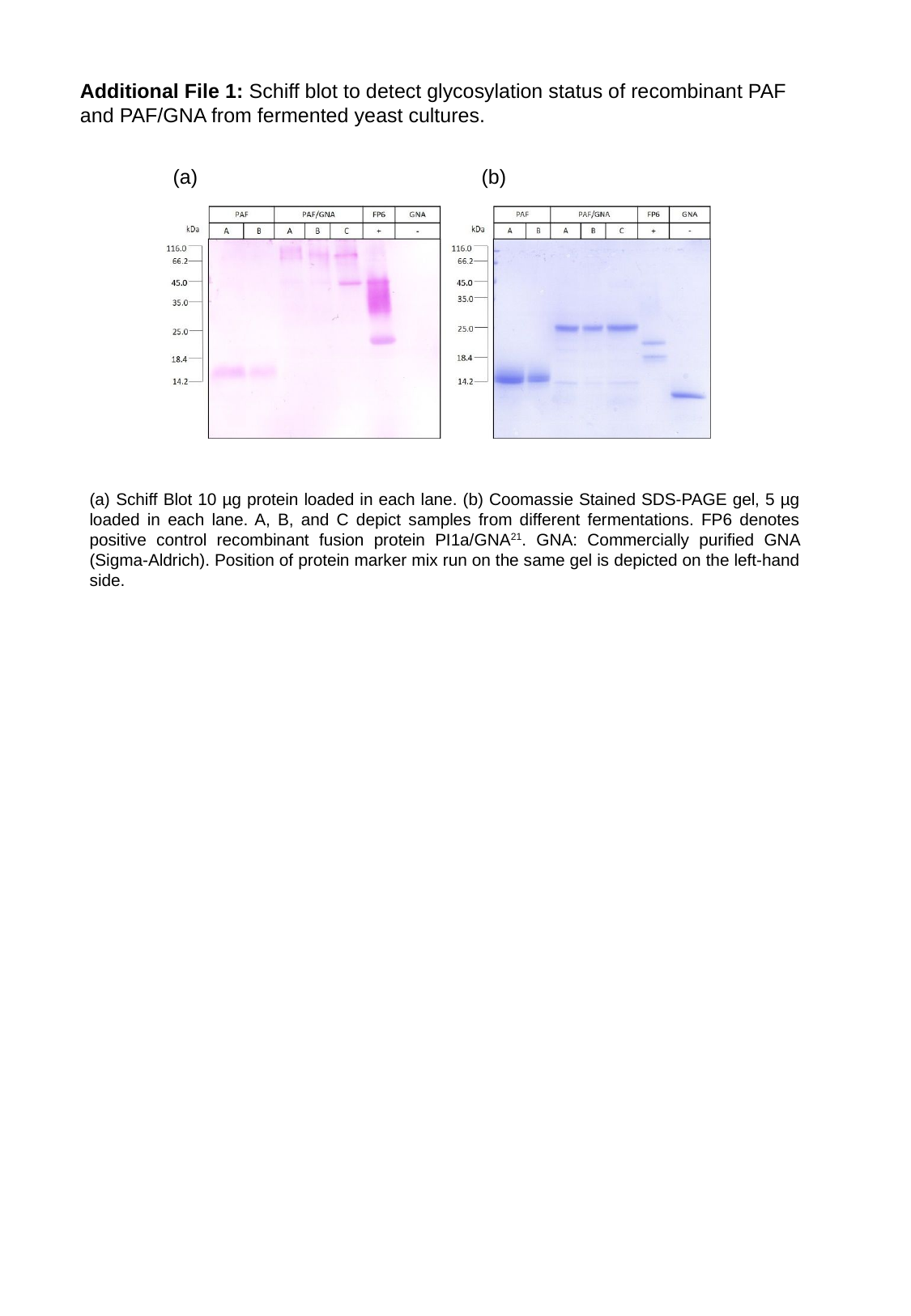

Additional File 1: Schiff blot to detect glycosylation status of recombinant PAF and PAF/GNA from fermented yeast cultures.
(a)
(b)
(a) Schiff Blot 10 µg protein loaded in each lane. (b) Coomassie Stained SDS-PAGE gel, 5 µg loaded in each lane. A, B, and C depict samples from different fermentations. FP6 denotes positive control recombinant fusion protein PI1a/GNA21. GNA: Commercially purified GNA (Sigma-Aldrich). Position of protein marker mix run on the same gel is depicted on the left-hand side.
