# Supplementary material for: Heterologous production of the insecticidal pea seed albumin PA1 protein by Pichia pastoris and protein engineering to potentiate aphicidal activity via fusion to snowdrop lectin Galanthus nivalis agglutinin; GNA)
Source: Microb Cell Fact. 2023 Aug 17;22:157. doi: 10.1186/s12934-023-02176-1 (PMC10436433; doi:10.1186/s12934-023-02176-1)
Supplement: Supplementary file 2 — Additional file 2: Primary structure of recombinant proteins - LC MS data Description: a) Primary structure of recombinant proteins expressed by transformed P. pastoris cells. Additional residues EAEAAA remain in expressed products due to incomplete processing of the alpha factor sequence by yeast dipeptidyl aminopeptidase, and the additional alanine is a consequence of gene insertion via a Pst I restriction site. The PA1b sequence is depicted in red, PA1a in blue, GNA in green and the linker region and histidine tags are in black. Remaining residues are the result of the cloning process for the expression construct. (b) LC-MS data obtained from ProAlanase and chymotrypsin digests of the PAF and PAF/GNA protein products. Blue bars depict identified peptides. [file 12934_2023_2176_MOESM2_ESM.pptx]

## Slide 1
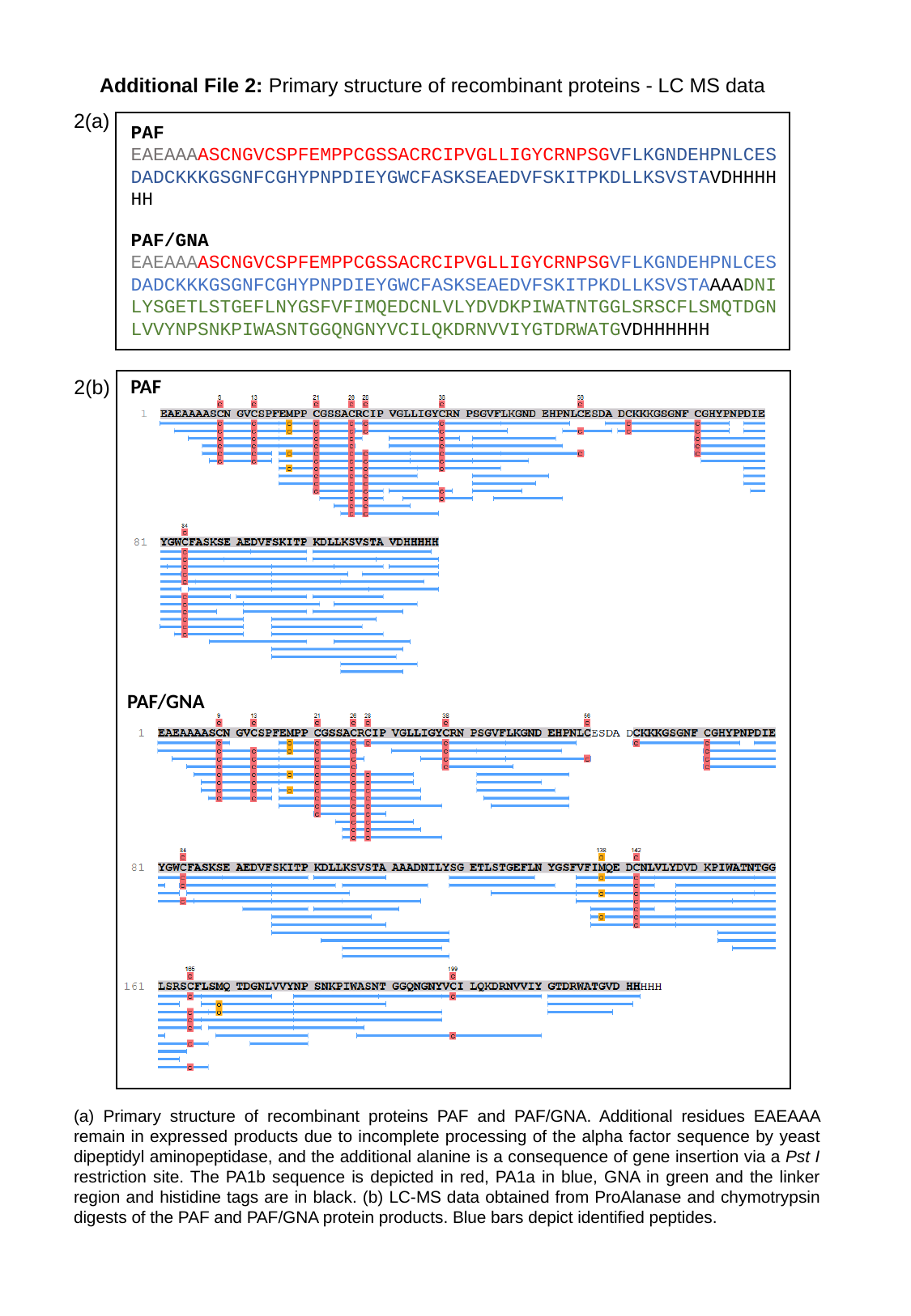

Additional File 2: Primary structure of recombinant proteins - LC MS data
2(a)
PAF
EAEAAAASCNGVCSPFEMPPCGSSACRCIPVGLLIGYCRNPSGVFLKGNDEHPNLCESDADCKKKGSGNFCGHYPNPDIEYGWCFASKSEAEDVFSKITPKDLLKSVSTAVDHHHHHH
PAF/GNA
EAEAAAASCNGVCSPFEMPPCGSSACRCIPVGLLIGYCRNPSGVFLKGNDEHPNLCESDADCKKKGSGNFCGHYPNPDIEYGWCFASKSEAEDVFSKITPKDLLKSVSTAAAADNILYSGETLSTGEFLNYGSFVFIMQEDCNLVLYDVDKPIWATNTGGLSRSCFLSMQTDGNLVVYNPSNKPIWASNTGGQNGNYVCILQKDRNVVIYGTDRWATGVDHHHHHH
PAF
PAF/GNA
2(b)
(a) Primary structure of recombinant proteins PAF and PAF/GNA. Additional residues EAEAAA remain in expressed products due to incomplete processing of the alpha factor sequence by yeast dipeptidyl aminopeptidase, and the additional alanine is a consequence of gene insertion via a Pst I restriction site. The PA1b sequence is depicted in red, PA1a in blue, GNA in green and the linker region and histidine tags are in black. (b) LC-MS data obtained from ProAlanase and chymotrypsin digests of the PAF and PAF/GNA protein products. Blue bars depict identified peptides.
