# Supplementary material for: Heterologous production of the insecticidal pea seed albumin PA1 protein by Pichia pastoris and protein engineering to potentiate aphicidal activity via fusion to snowdrop lectin Galanthus nivalis agglutinin; GNA)
Source: Microb Cell Fact. 2023 Aug 17;22:157. doi: 10.1186/s12934-023-02176-1 (PMC10436433; doi:10.1186/s12934-023-02176-1)
Supplement: Supplementary file 3 — Additional file 3: Examples of LC-MS spectra obtained following fragmentation of PAF and PAF/GNA proteins. Description: LC-MS spectra. [file 12934_2023_2176_MOESM3_ESM.pptx]

## Slide 1
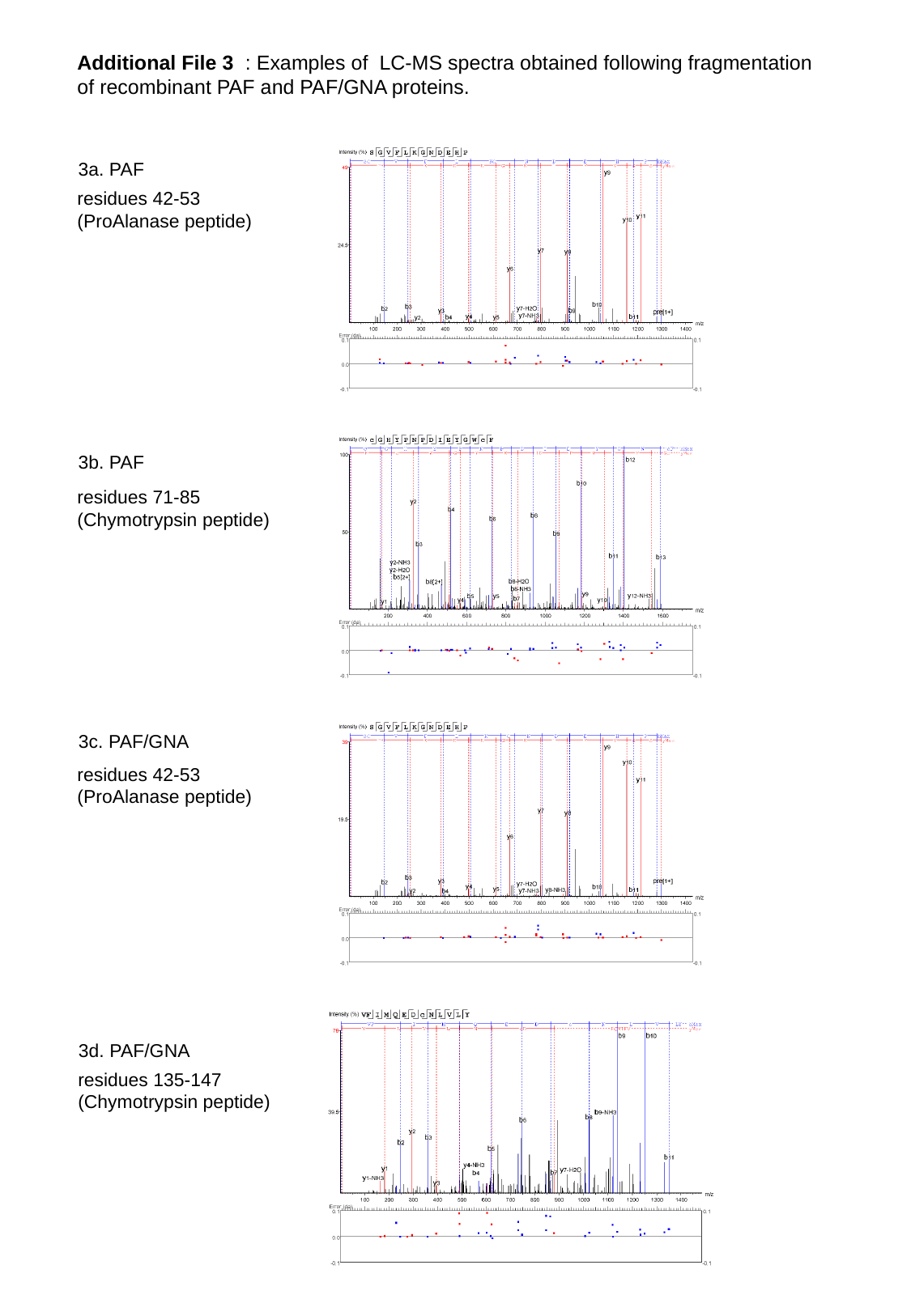

Additional File 3 : Examples of LC-MS spectra obtained following fragmentation of recombinant PAF and PAF/GNA proteins.
3a. PAF
residues 42-53
(ProAlanase peptide)
3b. PAF
residues 71-85
(Chymotrypsin peptide)
3c. PAF/GNA
residues 42-53
(ProAlanase peptide)
3d. PAF/GNA
residues 135-147
(Chymotrypsin peptide)
